# Surgical Correction of cicatricial Entropion in cattle: Two cases

**DOI:** 10.1002/ccr3.3755

**Published:** 2021-01-13

**Authors:** Caroline M. Betbeze, Meghan R. Villers, Mary R. Telle, Gretchen P. Grissett

**Affiliations:** ^1^ Mississippi State University College of Veterinary Medicine MSU MS USA

**Keywords:** bovine, cicatricial, entropion, Hotz‐Celsus, trichiasis

## Abstract

Utilization of the Hotz‐Celsus technique with standing sedation and local nerve blocks in cattle provides adequate correction of cicatricial entropion.

## Graphical abstract

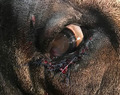

## 1 INTRODUCTION

An approximately 2‐year‐old Brahman bull and an approximately 6‐month‐old Angus cross steer presented to a veterinary teaching hospital with unilateral epiphora and blepharospasm. They were both found to be suffering from trauma‐induced cicatricial and spastic entropion which led to corneal ulceration in both cases. Due to the lack of response to tacking sutures and the appearance of the wounds, both received a Hotz‐Celsus repair. There are few reports of surgical entropion correction in cattle in the current veterinary literature. Utilization of the Hotz‐Celsus technique with standing sedation and local nerve blocks provided adequate correction of cicatricial entropion in both cases. Surgical repair is described in detail in order to provide a framework for practitioners to perform this surgery.

Entropion is inversion of the eyelids and is common in many species. It typically results in trichiasis, blepharospasm, and excessive lacrimation. This condition is rarely diagnosed in cattle.^1^ Limited reports of entropion correction in the bovine exist with variable levels of detail about surgical procedures and long‐term results.^1^, ^2^, ^3^ Potential causes of entropion include congenital, hereditary, cicatricial, spastic, and acquired. Common acquired causes are enophthalmos, dehydration, extraocular muscle atrophy, or phthisis bulbi.^1^, ^4^ Cicatricial entropion is typically caused by trauma and leads to fibrosis and scarring of the eyelid, commonly causing skin contraction and involution of the eyelid. Trichiasis results from the involution of the eyelid margin, often leading to ulcerative and nonulcerative keratitis. The underlying cause and severity of entropion typically influence the treatment chosen.

Correcting entropion is important in any species due to the discomfort for the animal and the potential to cause severe, persistent, or recurrent corneal ulcers which can lead to corneal perforation, severe corneal scarring, and loss of vision or the globe. Ocular pain caused by corneal ulceration can affect both production and reproduction in food animals because of decreased vision and comfort.^5^ For livestock, loss of vision or the globe can affect their production ability and sale price. Two visual eyes are required to pass a breeding soundness examination in a bull because a decrease in vision can lead to inadequate estrus detection within a herd. Sale price is often lower if an eye has been removed because buyers assume it was removed due to neoplasia, which is a reason for condemnation of a carcass.^6^ The cases reported here describe the presentation and successful treatment of cicatricial entropion in two unrelated bovines.

## 2 CASE 1

A 2‐year‐old Brahman bull presented to the Mississippi State University Animal Health Center for a 2‐week history of epiphora and blepharospasm in the right eye (OD). The client observed him rubbing his head on wooden posts in the pasture prior to the recognition of clinical signs. One week prior to presentation, the referring veterinarian diagnosed the bull with keratoconjunctivitis. The bull was administered two injections of oxytetracycline (Zoetis, Kalamazoo, MI) (20 mg/kg SQ q72h). After failure to respond to treatment, the bull was referred for further evaluation.

On presentation, vital parameters were within normal limits. Blepharospasm and excessive lacrimation were present in the right eye (OD). Periocular blepharedema was present OD. Vision was present in both eyes (OU) with normal pupillary light reflexes, palpebral reflexes, and menace responses.

**FIGURE 1:**
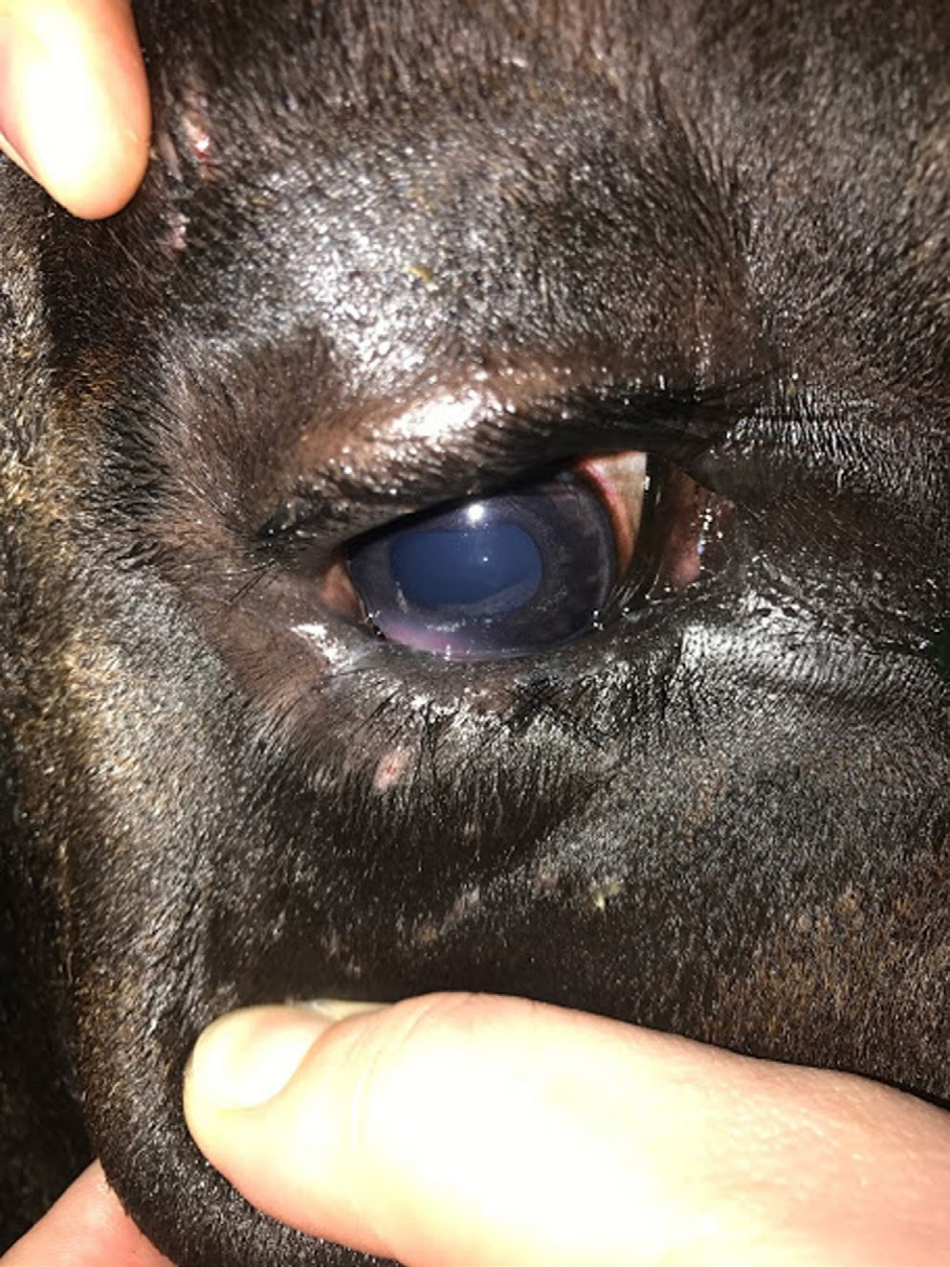
Entropion in the left eye of case 1. A white, vertical scar is noted 4 mm from the lateral canthus. Note that the eyelid margin is not visible, and there is excessive lacrimation, corneal edema, and vascularization of inferotemporal cornea

**FIGURE 2:**
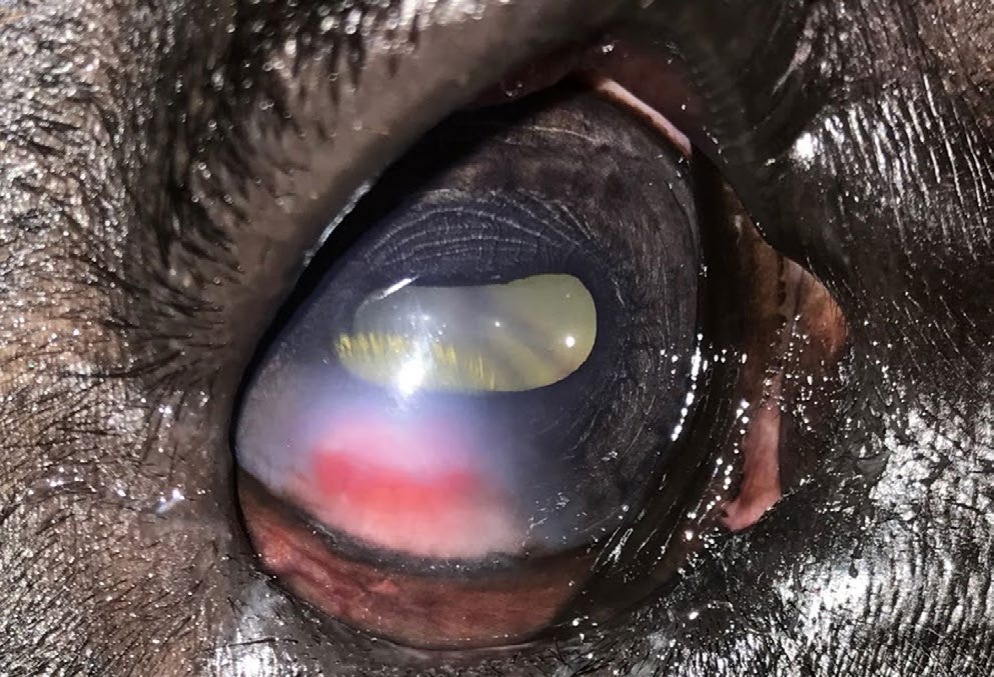
Epithelial corneal ulceration present in inferotemporal cornea with vascularization and granulation tissue

Proparacaine (Sandoz, Princeton, NJ) was applied OD to provide analgesia and facilitate a thorough ophthalmic examination. Complete resolution of the entropion after application of topical anesthetic did not occur, supporting that entropion was more than just spastic in nature. A thickened, scarred vertical segment of skin was noted at the lateral aspect of the inferior palpebral margin causing displacement of the eyelid margin toward the globe, which suggested cicatricial entropion. (Figure 1) Additionally, an epithelial, inferotemporal corneal ulcer OD measuring approximately 7 by 3mm in diameter was seen. This ulceration retained fluorescein stain. It was surrounded by corneal vascularization and corneal edema, but no signs of infection, such as cellular infiltrate, stromal involvement, or malacia, were noted. There was pale pink granulation tissue present on the ventral aspect presumptively due to chronicity and continuous irritation from trichiasis. (Figure 2).

The history and ophthalmic examination suggested that an eyelid wound had healed with scarring. This wound contracted, bringing the eyelid into closer association with the globe, which led to trichiasis causing a corneal ulcer. Entropion worsened due to severe blepharospasm and spastic entropion, which caused the corneal ulcer to persist.

After the initial examination, two horizontal mattress tacking sutures were placed using #2 Braunamid (Jorvet, Loveland, CO) in the inferior palpebra OD to retract the eyelid margins from the cornea to temporarily relieve trichiasis and allow the ulcer to heal. Atropine Sulfate 1% ophthalmic ointment (Akorn, Lake Forest, IL) was given every 24 hours for cycloplegia to control pain related to corneal ulceration and reflex uveitis. Triple antibiotic ophthalmic ointment containing bacitracin, neomycin, and polymyxin B (Bausch and Lomb, Tampa, FL) was started every 8 to 12 hours to provide broad‐spectrum antibacterial protection. A 1.1mg/kg dose of flunixin meglumine (Pfizer, New York, NY) was administered once intravenously for pain and inflammation. Epiphora and blepharospasm continued the next morning, indicating that the temporary tacking sutures were insufficiently retracting the eyelid.

After removal of the tacking sutures, a third eyelid flap was placed OD using #2 Braunamid to protect the ulcer from trichiasis. Atropine and triple antibiotic ointments were applied once prior to placement of the flap. Meloxicam (Boehringer Ingelheim, Ridgefield, CT) was started for continued pain management at a tapering dose of 1mg/kg PO every 24 hours for 3 days followed by 0.5mg/kg orally every other day for 3 doses. Oxytetracycline was given at 20 mg/kg subcutaneously on the day of presentation and day three of hospitalization.

**FIGURE 3:**
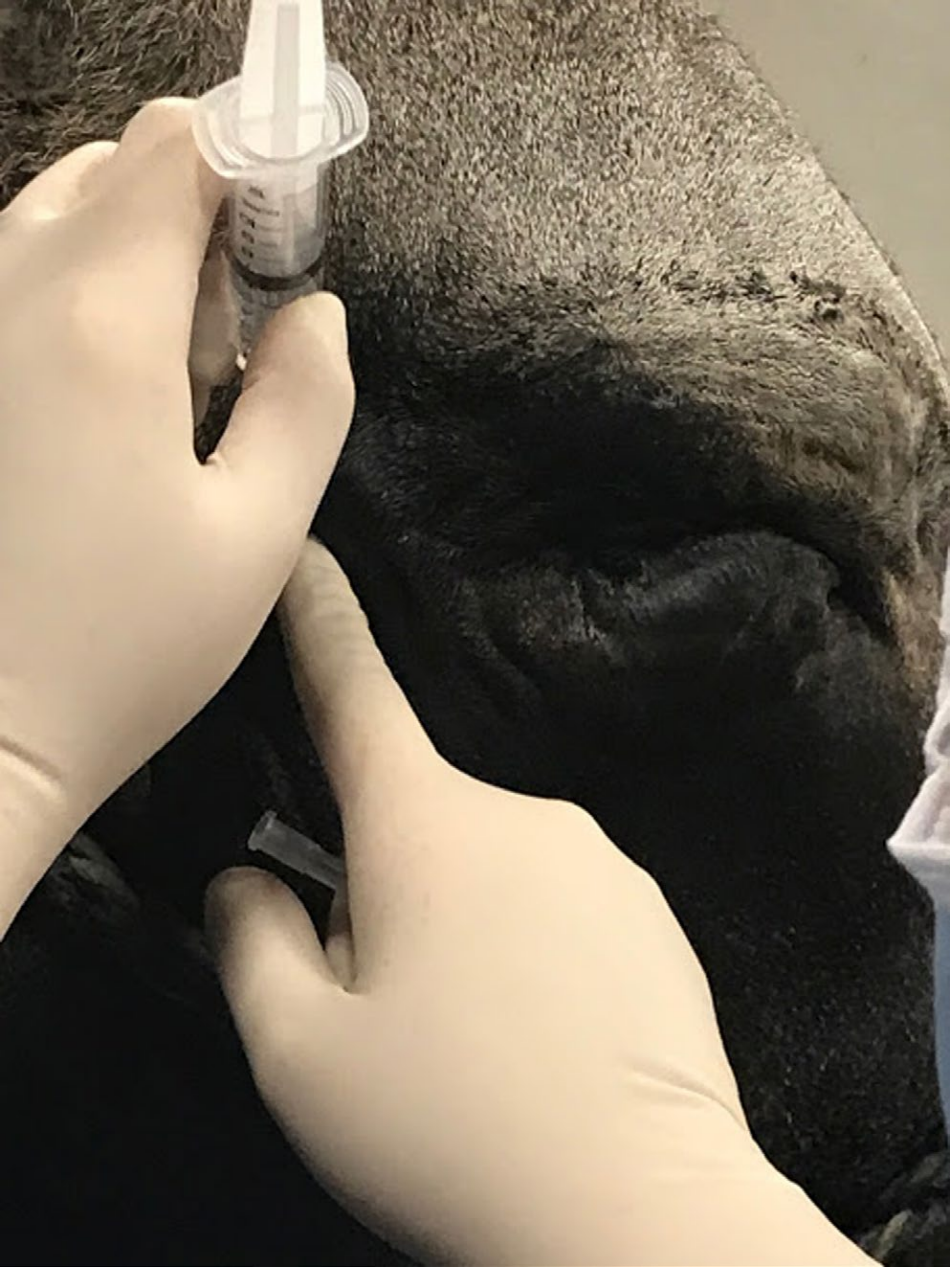
Auriculopalpebral nerve block prior to Hotz‐Celsus repair. Injection was performed midway between the base of the ear and the lateral canthus overlying the zygomatic arch

**FIGURE 4:**
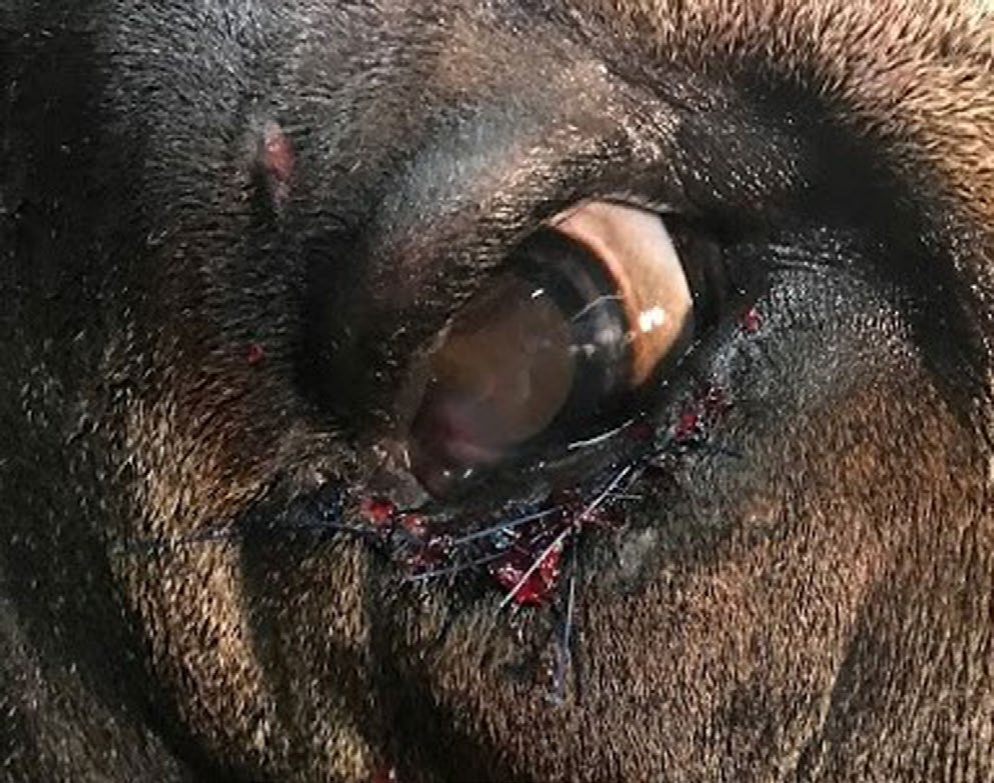
Completed Hotz‐Celsus repair OD. Note the suture tags are not contacting the cornea, and the eyelid margin is visible. No blepharospasm is noted

The third eyelid flap was removed one week later. Fluorescein stain defined the ulcer diameter as approximately 2 × 2 mm at that time. Because the ulcer and significant entropion were still present, standing sedation (0.01 mg/kg Butorphanol, 0.02mg/kg Xylazine, and 0.04mg/kg Ketamine administered intravenously) was used to perform a Hotz‐Celsus surgery on the inferior palpebra. The bull was placed in a hydraulic chute with halter tied to the head plate for restraint. The eyelashes were trimmed, and the eyelids were prepared with dilute (1:50) iodine solution and saline. An auriculopalpebral nerve block and a line block 2‐3 mm inferior to the eyelid margin were performed using 2% lidocaine (Hospira, Lake Forest, IL) to provide lack of sensation and akinesia of the right inferior eyelid (Figure 3). Proparacaine was applied to the corneal surface to provide analgesia. A Jaeger eyelid plate was placed in the inferior conjunctival fornix to stabilize the eyelid and protect the cornea. After measuring the segment of inferior palpebrae that had been touching the cornea with Jameson calipers, this segment of epidermis and superficial dermis, approximately 6 mm wide and 15 mm long, was removed, with the initial incision 3 mm inferior to the eyelid margin at the haired‐nonhaired junction. The ellipse was widest at the portion of the eyelid that was most inverted, tapering just past that portion on either end. The area suspected to be scar tissue was resected in this elliptical portion of skin. This width of resection was found to provide adequate retraction of the palpebral margin, so the resulting defect was closed using 0 nylon monofilament (Ethilon, Ethicon, Somerville, NJ) suture in a simple interrupted pattern. (Figure 4) A temporary tarsorrhaphy was placed with 0 PDS suture in a horizontal mattress pattern with stents (segments of extension set tubing) to prevent trichiasis from spastic entropion while the surgical site and corneal ulceration healed.

The temporary tarsorrhaphy was removed five days postoperatively. Upon removal, the corneal ulcer had healed and there was no evidence of trichiasis, indicating that the entropion was successfully corrected. The OD was comfortably open without excessive lacrimation. The sutures were removed nine days postoperatively.

## 3 CASE 2

A 6‐month‐old Angus cross steer presented for a swollen right eye with discharge along the palpebrae. Ophthalmic examination revealed a full‐thickness puncture wound to the inferior palpebra. The cornea was negative for fluorescein stain uptake, and the wound was healing well by second intention. Neomycin, bacitracin, and polymyxin B ophthalmic ointment was administered OD every 12 hours to provide topical antimicrobial coverage to the conjunctival aspect of the wound. Fly repellent was applied daily. Oxytetracycline (20 mg/kg) was administered subcutaneously once.

One week later, the wound had healed, but cicatricial entropion of the inferior palpebra was present. Three temporary tacking sutures were placed to externally rotate the eyelid margin, with the goal of causing scarring that would lead to permanent rotation. The sutures were removed ten days after placement because tissue reaction of the subcutaneous tissues felt adequate to ensure appropriate scarring. The entropion immediately recurred after removal of tacking sutures.

A Hotz‐celsus procedure was performed on the inferior palpebra. For this procedure, the patient was heavily sedated (0.05 mg/kg Xylazine, 0.025 mg/kg Butorphanol, 0.1 mg/kg Ketamine intravenously) and placed in lateral recumbency. A line block with 2% lidocaine (Hospira, Lake Forest, IL) was performed along the proposed incision. An elliptical segment of epidermis and superficial dermis, approximately 4 mm in diameter, was removed from the inferior palpebra just ventral to the eyelid margin. The resulting defect was closed using 0 PDS suture in a simple interrupted pattern. No tarsorrhaphy was performed because corneal ulceration was not present and there was no evidence of spastic entropion. Sutures were removed two weeks postoperatively with no recurrence noted.

## 4 DISCUSSION

The cases presented here provide detailed examples of clinical management of cattle with entropion. Both cases had cicatricial entropion that occurred from previous injury to the eyelid which has not been reported in this species. In both cases, temporary tacking and relief of spastic entropion did not correct the underlying problem, necessitating permanent surgical repair. Because entropion is rarely reported, there is little comprehensive information available on surgical options in cattle.^1^, ^2^, ^3^ The reports in the literature are single cases without much detail in journals that are difficult to obtain.

There are important considerations when selecting an entropion repair technique in any species. If a corneal ulcer is present, it is preferable to temporarily resolve spastic entropion prior to surgical repair to avoid the risk of creating ectropion through overcorrection.^4^ Another reason to perform temporary correction is to allow the animal to reach maturity before performing conformational entropion correction.^4^ In both of our cases, the underlying cause was determined to be scar tissue; however, case 2 was only 6 months old at the time of presentation. Because of the presence of a corneal ulcer in case 1 and young age in case 2, temporary techniques, such as tacking sutures and third eyelid flap, were employed to encourage resolution of spastic entropion and potentially prevent the need for surgical repair. The corneal ulcer of case 1 did not heal despite use of tacking sutures and third eyelid flap. In case 2, the entropion immediately recurred after removal of the tacking sutures. After the Hotz‐celsus procedure, ectropion was not noted in either case even though spastic entropion was still present prior to permanent repair. This suggests that the Hotz‐Celsus can be employed initially in cases of cicatricial entropion in cattle to prevent morbidity. The surgeon must recognize the presence of spastic entropion and assess the amount of skin to be removed with the knowledge that both types of entropion are present.

The Hotz‐Celsus technique is the most common surgery used for permanent repair of entropion. This technique involves removal of an elliptical portion of skin adjacent to the eyelid margin in order to roll the eyelid away from the cornea. In the past 10‐15 years, wedge resection combined with the Hotz‐Celsus or lateral canthal closure techniques have been recommended in canine and feline entropion to reduce the likelihood of overcorrection or need for a second surgery.^4^, ^7^, ^8^ Lateral wedge resection is commonly used with euryblepharon or lower lateral entropion to shorten and remove the section that is causing irritation.^4^ This technique has also been used successfully to repair congenital entropion and euryblepharon in 5 Boer goat kids.^9^ Wedge resection or lateral canthal closure was deemed unnecessary for these cases because the cause for entropion was scar tissue and the eyelid was a normal length. Regional nerve blocks were employed in these cases to provide akinesia and analgesia in order to perform surgery in these heavily sedated bovines. Local eyelid blocks are often avoided in small animals undergoing blepharoplasty because of the potential to alter the surgical area causing possible overcorrection. Because these blocks are needed in cattle, it is important to make a surgical plan prior to injection in order to avoid miscalculation of the amount of skin to be removed.

There are many suture choices for entropion repair; polyglactin, nylon, and silk have all been recommended.^4^ In small animals, 4‐0 to 6‐0 suture with a swaged‐on cutting needle is suggested as the best choice.^4^ In previous reports in cattle, 3‐0 polyglactin and nylon of an unspecified size were used with success.^1^, ^2^ In our cases, 0 nylon and 0 polydioxanone (PDS) were used for the Hotz‐Celsus repair. This larger size seemed appropriate for full‐grown cattle and helped to control the spastic portion of the entropion; however, a smaller size may be suitable for younger and smaller cattle. Nylon is nonreactive and commonly used for skin but is nonabsorbable and needs to be removed. Even though PDS is absorbable, it is stiff and will not dissolve quickly. It is possible that cattle may rub and cause postoperative trauma with this rigid suture type. In cattle, sutures with tags cut very short or intradermal sutures could be considered so that they do not need to be removed later. In case 1, which had a corneal ulcer, a lateral temporary tarsorrhaphy was also placed following the Hotz‐Celsus in order to prevent trichiasis while healing. The corneal ulcer healed while the tarsorrhaphy was in place.

Major considerations for entropion correction in cattle are the ability to safely handle the animal for surgery and ability to give medications. This is affected by the accessibility of the animal on the farm, the veterinary/care facilities available, and availability of skilled personnel. The cases presented here were hospitalized at a teaching hospital, allowing for safe medication administration and close monitoring. This was a major advantage that likely contributed to success. Both cases healed within 7‐10 days.

Injections of various substances have been reported for entropion repair in sheep, dogs, and cats.^4^, ^6^, ^10^ Subcutaneous injections of sterile air or water, procaine penicillin or oxytetracycline, or mineral oil into the inferior palpebra have been reported for congenital entropion in lambs.^4^, ^6^, ^11^ This alters the eyelid margin alignment by causing inflammation and fibrosis in the inferior palpebra to rotate the margin superficially.^6^, ^11^ Injection of hyaluronic acid, a subdermal filler, has recently been reported as a treatment for dogs and cats with entropion.^10^ These injections are easy to perform and do not require anesthesia or sedation. Good long‐term results have been reported with little postinjection care. These methods were not considered for our cases due to the severity of the entropion and the presence of scar tissue but could potentially be used for milder cases in cattle that are unable to have follow‐up care.

Both animals in this case report healed with little morbidity after their repair. The entropion was not overcorrected even though spastic entropion and corneal ulceration were still present prior to permanent surgery. There were no postoperative complications, and the entropion has not recurred in either case in the year since treatment.

## 5 ETHICS STATEMENT

We confirm that the manuscript was read and approved by all authors. We confirm that the order of authors has been approved by all authors and that no other authors satisfied the criteria for authorship. We confirm that we have given due consideration to the protection of intellectual property associated with this work and that there are no impediments to publication. All owners signed informed consent for procedures deemed necessary upon hospitalization for the animals in this case series.

## CONFLICT OF INTEREST

There are no conflicts of interest for any authors for this manuscript.

## AUTHOR CONTRIBUTIONS

Meghan R. Villers: was the student assigned to the Brahman bull case and originally wrote the case report on the bull for a school project; Gretchen P. Grissett: was the clinician in charge of both cases, wrote the case of the 6‐month‐old bull, and edited the manuscript; Mary R. Telle: provided vital editing of manuscript and research on appropriate journals for case reviews; Caroline M. Betbeze: is the corresponding author, consulted on both cases, performed the surgery on the Brahman bull, wrote the discussion portion of the manuscript, provided extensive editing of manuscript, and submitted the manuscript.
